# A Comparative UPLC/HRMS Molecular Networking-Enhanced Study on the Phenolic Profiles and Bioactivities of Three Medicinally Significant Species of *Onosma* (Boraginaceae)

**DOI:** 10.3390/plants13243468

**Published:** 2024-12-11

**Authors:** Evgenia Panou, Gokhan Zengin, Nikola Milic, Christos Ganos, Konstantia Graikou, Ioanna Chinou

**Affiliations:** 1 Laboratory of Pharmacognosy and Chemistry of Natural Products, Department of Pharmacy, National & Kapodistrian University of Athens, Zografou, 15771 Athens, Greece; evpanou@pharm.uoa.gr (E.P.); nmilic@pharm.uoa.gr (N.M.); cganos@pharm.uoa.gr (C.G.); kgraikou@pharm.uoa.gr (K.G.); 2 Laboratory of Physiology and Biochemistry, Department of Biology, Science Faculty, Selcuk University, 42130 Konya, Turkey; gokhanzengin@selcuk.edu.tr

**Keywords:** *Onosma leptantha*, *O. graeca*, *O. erecta*, aerial parts, Boraginaceae, phenolic compounds, UPLC/HRMS, feature-based molecular network, antioxidant activity, enzyme inhibition

## Abstract

The current work represents a comparative study of the phenolic profiles of three under-explored *Onosma* (Boraginaceae) species from Greece—*Onosma leptantha* (OL), *Onosma erecta* (OE), and *Onosma graeca* (OG). Although *Onosma* spp. have ethnopharmacological significance, previous phytochemical studies have focused primarily on roots. Methanolic extracts of the aerial parts were analyzed using qualitative LC-MS enhanced by molecular networking-based dereplication, annotating 94 phenolics categorized into hydroxybenzoic acids (7), hydroxycinnamic acids (24), lignans (14), neolignans (14), stilbenes (4), coumarins (5), and flavonoids (26). OG exhibited the broadest distribution of flavonoid glycosides. OL contained the greatest number of hydroxycinnamic and neolignan derivatives, and OE was notably abundant in lignans. Total phenolic (TPC) and total flavonoid (TFC) contents were quantified, and the antioxidant capacity and enzyme inhibition against cholinesterases, α-amylase, and α-glucosidase were assessed. OL showed a high TPC (69.03 mg GAE/g extract) and strong antioxidant activity, while OG exhibited a high TFC (45.80 mg RE/g extract). All extracts demonstrated stronger AChE inhibition than BChE, with OG showing the highest AChE inhibition (2.35 mg GALAE/g). Additionally, OL was the most active against both α-glucosidase (5.69 mmol ACAE/g) and α-amylase (0.48 mmol ACAE/g). This study improved our understanding of the chemical diversity within these species, providing a more comprehensive insight into their longstanding ethnopharmacological potential.

## 1. Introduction

Boraginaceae is a family of trees, shrubs, and perennial or annual herbs with widespread distribution [1]. The family encompasses about 150 genera and 3500 species [2] thriving particularly in Mediterranean climates, but is also prevalent in diverse habitats across the continents [1]. Pyrrolizidine alkaloids (PAs) and naphthoquinones are the most striking secondary metabolites that the Boraginaceae family is known for. PAs have received considerable interest as a class of potentially toxic natural products due to their association with hepatotoxicity. The isomeric red pigments alkannin and shikonin, found typically in the roots, are the main representatives of quinonoid compounds with multiple pharmacological properties ranging from wound healing to anti-inflammatory, antimicrobial, antitumor, and antiviral [3,4]. The family is also a rich source of phenolic compounds, such as flavonoids (mostly glycosides of flavonols and flavones) and hydroxycinnamic acid derivatives, with caffeic and rosmarinic acids being two of the most ubiquitous metabolites [1,4]. Numerous metabolites originating from phenylpropanoid metabolism, including lignans and neolignans, have also been identified within the family [1].

The genus *Onosma* is one of the largest among the genera of the Boraginaceae family [5], with the Asian continent having the highest share in terms of its species diversity, most of which are distributed in Turkey [6]. In Greece, the genus is represented by 20 species, 13 of which are endemic [7]. The etymology of the name *Onosma* derives from the Greek word *osmê* (ὀσμή, f.), which is Latinized as *-osma* (f.) and was introduced into modern botanical nomenclature by Linnaeus, most probably referring to the species known for their distinctive scents [8]. *Onosma* species thrive in dry or moist, sunny environments, typically in rock crevices, and are commonly referred to as rock garden plants [9]. Some ethnobotanical studies have revealed their history in medicinal uses as laxatives, tonics, and remedies to treat heart and kidney disorders, bronchitis, abdominal pain, and fever [5,6]. Most specifically, root preparations were used topically for wound healing, burns, and to treat skin eruptions, while flowers were used as stimulants in the treatment of rheumatism and palpitations of the heart [9]. Leaf powder has also been given to children as a purgative [9]. Other pharmacological studies have shown that their extracts and phytochemicals exhibit a variety of therapeutic properties, including antioxidant, enzyme-inhibitory, antitumor, hepatoprotective, antiviral, anti-inflammatory, and antimicrobial [6].

In the current study, methanolic extracts of the aerial parts of *Onosma leptantha* Heldr. (OL), *Onosma erecta* Sibth. and Sm. (OE), and *Onosma graeca* Boiss. (OG) collected from southern Greece were chosen for analysis, with the primary aim of evaluating and comparing their phenolic profiles. OG is distributed in southern Europe, particularly in Greece, while OL and OE are both endemic [7]. They are perennial herbs featuring lanceolate leaves and yellow flowers, growing in dry, rocky environments [10].

The aerial parts of OG and OE have already been analyzed in our laboratory for their alkaloid content, resulting in the isolation of several major PAs, leptantine N-oxide, onosmoleptine N-oxide, echihumiline N-oxide, 7-O-acetylechinatine N-oxide, onosmerectine N-oxide, and 7-epiechimiplatine N-oxide, and one stereoisomer of viridinatine N-oxide [10,11]. Root extracts of OE and OG have also been examined for their naphthoquinone content, resulting in the isolation of deoxyalkannin, propionylalkannin, acetylalkannin, β-hydroxyisovalerylalkannin, and *β*,*β*-dimethylacrylalkannin, but solely for OG [3].

The previous investigations into the chemical composition of the aerial parts of *Onosma* species have demonstrated a significant presence of phenolic compounds [9]. These phenolic compounds are well known for their association with various biological activities, particularly their potent antioxidant properties [12]. They function as reducing agents, hydrogen donors, and metal chelators, thereby inhibiting the formation of free radicals [4]. Numerous physical ailments are linked to oxidative stress and the overproduction of free radicals [13]; consequently, extracts with an elevated phenolic content have garnered considerable research interest in recent years.

Numerous plants serve as sources of compounds that exhibit inhibitory effects on the enzymes involved in essential metabolic processes, and several of these enzymes are considered significant targets in the management of chronic diseases. Acetylcholinesterase (AChE) and butyrylcholinesterase (BChE) are the critical enzymes implicated in *β*-amyloid accumulation, a process associated with Alzheimer’s disease and other neurodegenerative disorders [4]. Furthermore, α-amylase and α-glucosidase are the key enzymes in carbohydrate metabolism and important targets for therapeutic interventions aimed at postprandial hyperglycemia, and consequently type-2 diabetes [14]. The literature indicates that *Onosma* species demonstrate a notable inhibitory effect against the aforementioned enzymes, with phenolic compounds identified as the primary constituents responsible for this activity [15].

As a continuation of our research on Greek *Onosma* species, we present a comparative study of the phenolic profiles of three species, noting that they have not previously been analyzed in this context. In this investigation, we conducted a comparative analysis of the chemical profiles using the UPLC-HRMS technique, enhanced by a contemporary computational mass spectrometry approach and feature-based molecular networking (FBMN) dereplication. Additionally, we conducted a series of quantification tests and biological assays on the yielded crude extracts, namely, the total flavonoid and total phenolic contents, antioxidant activity, as well as the enzyme-inhibitory effects (AChE and BChE, α-amylase, and α-glycosidase), taking into consideration that there are no previous reports regarding these biological activities of the selected species.

## 2. Results

### 2.1. UPLC-HRMS Analysis

**Figure 1:**
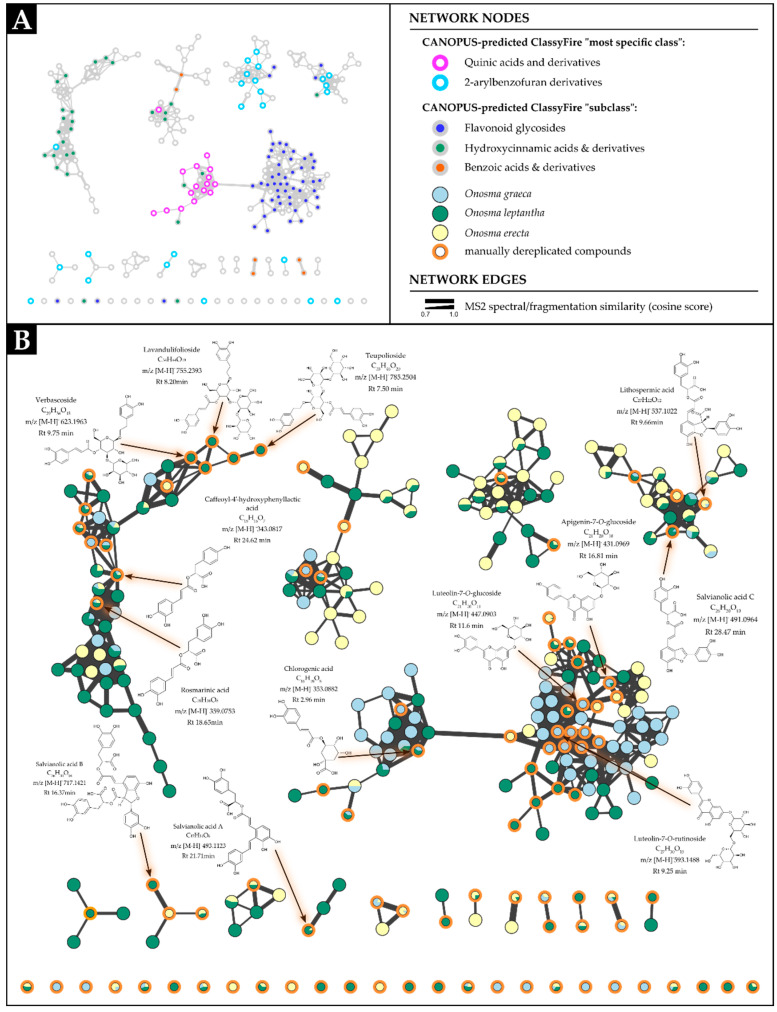
Feature-based molecular networking (FBMN) of the methanolic extracts of three Greek *Onosma* (Boraginaceae) species, based on data acquired in ESI (-) mode, visualized in Cytoscape. (**A**) The FBMN includes the superimposed results from the ClassyFire chemical classifications predicted by CANOPUS (prediction probability threshold ≥ 0.7). (**B**) A customized version of the same FBMN illustrates the distribution of features across the examined species, highlighting several characteristic compounds that were manually dereplicated. Please refer to the figure legend for explanations regarding the colors and shapes of the nodes and edges.

The UPLC-HRMS analysis, which integrated both manual and computational approaches (Table 1, Figure 1), resulted in the annotation of 94 metabolites in total, across the three studied *Onosma* species. The GNPS2 workflow utilized, led to the construction of an FBMN, consisting of 1166 nodes (aligned consensus MS2 spectral features from all three species) and 702 edges (connections between the nodes based on MS2 spectral/fragmentation similarities) (Supplementary Materials Figure S2). By superimposing the CANOPUS output of chemical classification predictions onto the FBMN within the Cytoscape environment, integration and refinement of the results, focusing on compound classes of interest (i.e., phenolics) was possible, allowing for further analysis and interpretation. The nodes annotated via the aforesaid approach exhibited a clustering pattern that reflected, for the majority of the nodes, their distinct chemical class (expressed as ClassyFire “chemical subclass” or “most specific class”, with a probability threshold greater than 0.7). Furthermore, on closer inspection, this clustering pattern also revealed an intrinsic relationship between the connected features based on their biosynthetic origin [12].

The above led to the annotation of the phenolic compound clusters into seven phenolic subgroups based on their predominant structural moiety and their biosynthetic origin, as follows: (a) hydroxybenzoic acid derivatives (7 compounds), (b) hydroxycinnamic acid derivatives primarily consisting of caffeic and coumaric acid derivatives (24 compounds), (c) neolignans, specified as 2-arylbenzofuran derivatives (14 compounds), (d) lignans specified as arylnapthalene or dihydroarylnapthalene derivatives (14 compounds), (e) stilbene derivatives (4 compounds), (f) coumarins (5 compounds), and (g) flavonoids and flavonoid glycosides, which encompassed flavone derivatives of apigenin, luteolin, or diosmetin, as well as flavonol derivatives of kaempferol, quercetin and isorhamnetin (26 compounds).

### 2.2. Total Phenolic Content (TPC) and Flavonoid Content (TFC)

**Figure 2:**
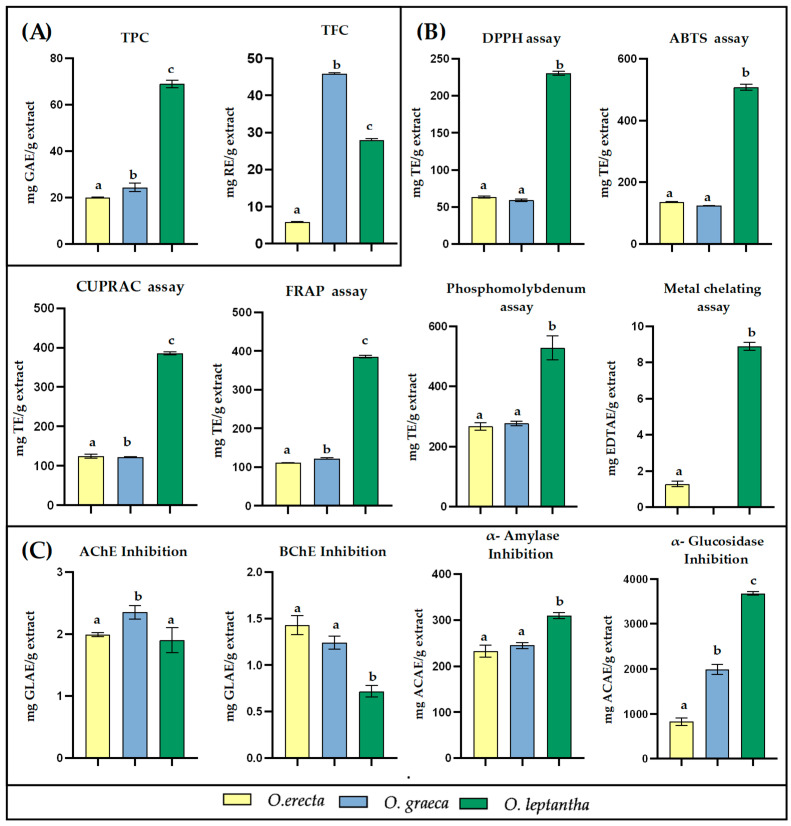
(**A**) Total phenolic content (TPC) and Total flavonoid content (TFC), (**B**) Antioxidant assays, (**C**) Enzyme inhibition assays for three *Onosma* methanolic extracts. Values indicated by different superscripts in each assay indicate significant difference (*p* < 0.05).

Total phenolic and flavonoid contents were evaluated in the methanolic extracts of the aerial parts of the three examined *Onosma* species and the results are presented in Table 2, Figure 2A.

### 2.3. Biological Assays

#### 2.3.1. Antioxidant Activity

The antioxidant activities of the methanolic extracts of *Onosma* species were assessed using different assays, particularly DPPH•, ABTS•+, CUPRAC, FRAP, phosphomolybdenum and a metal (ferrous ion) chelating test. The results of the assays are presented in Table 3, Figure 2B.

#### 2.3.2. Enzyme-Inhibitory Activity

The enzyme-inhibitory effects of the *Onosma* species were tested against different enzymes, including acetylcholinesterase (AChE), butrylcholinesterase (BChE), α-amylase and α-glucosidase, and the results are summarized in Table 4, Figure 2C.

#### 2.3.3. Correlation Coefficients Between the Assays

In order to establish a potential correlation between the TPC and TFC and the biological activity of the studied extracts, the results obtained from all the assays were subjected to a statistical analysis (Table 5).

## 3. Discussion

### 3.1. Phytochemical Analysis of Onosma Species

The comparative study of the phenolic profiles of the three investigated *Onosma* spp. using computational and manual dereplication approaches led to the annotation of 94 phenolic compounds (59 from OL, 46 from OE, and 43 from OG). The output generated from MZmine processing underwent a meticulous manual dereplication analysis, which involved a fragmentation study, by comparing the obtained MS2 spectra with the data existing in the phytochemical literature on *Onosma* spp. and the Boraginaceae family. Concurrently, the MZMine-derived features were utilized to construct an FBMN using the GNPS2 platform and to make CANOPUS-based predictions within the SIRIUS environment. This process led to tentative annotations based on spectral library matches and chemical class predictions, respectively. Additionally, the manual dereplication approach was expanded to include selected unannotated nodes/features identified in the clustering data generated by the computational methods, thereby increasing the overall number of annotations.

As seen in Figure 1, within the FBMN, the majority of phenolic compounds formed distinct clustering patterns based on their MS2 spectral/fragmentation similarities expressed through cosine similarity coefficient above 0.7. CANOPUS predictions have introduced an additional dimension of clustering tendency among the features within the constructed FBMN, showing that phenolic compounds that share similar structural traits are congruent with the explained clustering patterns (Figure 1A).

Flavonoids and flavonoid glycosides were identified in all examined extracts, while the latter formed the most prominent subgroup within the molecular network (Figure 1A). Among the species studied, OG exhibited the highest share of flavonoid compounds, followed by OL, while OE contained the lowest levels of these metabolites. The TFC measured in the extracts with resulting values of 5.82, 45.80, and 27.94 mg RE/g extract for OE, OG and OL, respectively, is in accordance with this observation.

The dereplication process revealed a higher number of flavone (such as luteolin, apigenin, and diosmetin) derivatives in OG, while OE and OL contained more flavonol (kaempferol, quercetin, and isorhamnetin) derivatives. Notably, all studied species contained both apigenin and luteolin derivatives. These were identified mostly in OE and OG and they are among the most significant metabolites of the *Onosma* genus. They have been reported in numerous species, including *O. isaurica*, *O. bracteosa*, *O. lycaonica*, *O. papillosa*, *O. gigantea*, *O. pulchra*, *O. frutescens*, *O. aucheriana*, *O. sericea*, *O. mollis*, *O. inexspectata*, and *O. armenum* [6]. Their mono-glycosides, apigenin-7-*O*-glucoside and luteolin-7-*O*-glucoside, along with several respective *O*-hexosides, have been detected in all three studied species. In contrast, the diglycosides of apigenin and luteolin (such as apigenin-*O*-hexose-*O*-deoxyhexoses, luteolin-7-*O*-hexose-*O*-deoxyhexose, and luteolin-7-*O*-rutinoside) were found exclusively in OG, while luteolin acetyl-dihexoside was identified only in OL. The 7-*O*-glucosides of apigenin and luteolin are commonly reported in various species within the genus, along with their aglycones [6]. However, diglycosides are less prevalent; for instance, apigenin-*O*-rhamnosylhexoside has been reported only in extracts of *O. sericea* and *O. stenoloba* [25], while a luteolin-6,8-*C*-diglycoside has been only detected in *O. heterophylla* [49] before. Moreover, the presence of luteolin *O*-diglycosides such as luteolin-7-*O*-hexose-*O*-deoxyhexose, luteolin-7-*O*-rutinoside, and luteolin acetyl-dihexoside is identified for the first time within the *Onosma* genus.

The flavone diosmetin and its rhamnosylglucoside, diosmin, were exclusively identified in OG, while a trihydroxy-methoxyflavone dihexoside and a trihydroxy-methoxyflavone acetyl-dihexoside were detected only in OL. Diosmetin has been previously reported solely in the *O. sericea* [25], whereas diosmin has been found in both *O. sericea* and *O. heterophylla* [25,49]. The annotations of trihydroxy-methoxyflavone dihexoside and its acetyl-dihexoside are also documented for the first time in the *Onosma* genus.

Flavonols, including quercetin, kaempferol, and isorhamnetin, were predominantly identified in their glycosides’ forms. Specifically, quercetin-3-*O*-galactoside (hyperoside), quercetin-3-*O*-glucoside (isoquercetin), quercetin-3-*O*-rutinoside (rutin), and one quercetin-*O*-hexoside-*O*-deoxyhexoside were detected in both OE and OL. In contrast, OG contained only two distinct quercetin*-O*-hexoside-*O*-deoxyhexosides. Hyperoside is recognized as one of the most commonly reported metabolites within the genus, having been reported in the aerial parts of numerous species, including *O. isaurica*, *O. bracteosa*, *O. lycaonica*, *O. papillosa*, *O. pulchra*, *O. frutescens*, *O. aucheriana*, *O. sericea*, *O. trapezuntea*, *O. rigidum*, *O. mollis*, *O. inexspectata*, and *O. armenum* [6]. Additionally, another quercetin-*O*-hexoside has been documented in *O. sericea* and *O. stenoloba* [25], whereas rutin has been previously reported in *O. stellulata* [50]. Kaempferol glycosides were identified in OE and OL, while no kaempferol derivatives were detected in OG. Kaempferol-3-*O*-galactoside (trifolin) and kaempferol-*O*-hexose-*O*-deoxyhexosides were present in both OE and OL, whereas kaempferol 3-*O*-glycoside (astragalin) was found exclusively in OE. Kaempferol-*O*-hexosides have been reported in *O. sericea* and *O. stenoloba* [25] and two kaempferol rhamnoglycosides (kaempferol 3*-O*-[α-L-rhamnopyranosyl(1→2)-*β*-D-glucopyranoside, and kaempferol 3-*O*-[α-L-rhamnopyranosyl-(1→6)-*β*-D-glucopyranoside) have been isolated from the aerial parts of *O. bracteatum* [32], while one kaempferol 3-*O*-rhamnoside has been detected in *O. heterophylla* [49]. Isorhamnetin derivatives were exclusively identified in the extract of OL, where a dihexoside and an acetyl dihexoside were detected. In the *Onosma* genus, only isorhamnetin rhamnoglycosides have been reported in *O. stenoloba* and *O. stellulata* [25,50], marking these two derivatives new for the genus.

In the current study, hydroxycinnamic acids and their derivatives represent the second-largest chemical subclass of phenolics identified. p-Coumaric acid, caffeic acid, and its methyl ester were detected in all three species, along with rosmarinic acid and its methyl ester and hexosides. Salvianic acid A (danshensu) was detected in OE and OL. According to the literature, p-coumaric, caffeic, and rosmarinic acids are among the most widely distributed compounds within *Onosma* genus [6]. Salvianic acid A, methyl caffeate and rosmarinic acid hexosides have been identified before in *O. sericea* and *O. stenoloba* [25], while methyl rosmarinate has been reported in *O. sericea* and *O. bracteatum* [25,32].

Another category of phenolic metabolites detected in the examined plants is the one of 2-arylbenzofurans which are considered neolignan derivatives [51]. The majority of these metabolites were associated with OE and OL. The annotated metabolites of this category were tournefolic acid A, salvianolic acid C, lithospermic acid, and salvianolic acid B (lithospermic acid B), along with their esters (e.g., methyl lithospermate B) and glycosides (e.g., salviaolic acid B and C hexosides). In a recent investigation on phenolics from *O. heterophylla*, salvianolic acids B and C have been identified [49]. Furthermore, metabolites of 2-arylbenzofuran type have been detected so far in several genera within the Boraginaceae family such as *Tournefortia*, *Lithospermum*, *Alkanna*, and *Rindera* [4,29,52,53]. To the best of our knowledge, this study represents the first documented identification of methyl lithospermate B, tournefolic acid A, and glycosides of salvianolic acids B and C, within the genus *Onosma*. Some of these metabolites such as salvianolic acid B exhibit significant pharmacological activities, including antioxidant, antiplatelet, and anti-ischemic properties as well as protective effects on cardiovascular, and metabolic diseases, neurodegenerative disorders, and lung injuries [54].

Several lignan derivatives of the arylnapthalene and dihydroarylnapthalene subtype were also detected in the studied plant species. The compounds globoidnan B, 9′-methoxyl salvianolic acid R, and rabdosiin, along with its isomer and salts were predominantly identified in OE and OL. Trigonotins A, B, and C along with their isomers and globoidnan A were primarily detected in OG and OE. Pulmonarioside C, another lignan glycoside derivative, was also observed in OL. Some of these lignans, consisting of caffeic acid trimers or tetramers, have been already reported in the *Onosma* genus; specifically, globoidnan A has been identified in *O. bourgaei* and *O. bracteatum* [32,55], while 9′-methoxyl salvianolic acid R and pulmonarioside C were isolated from *O. bracteatum* [32]. Additionally, the p-coumaric acid ester of trigonotin A has been identified in *O. bracteatum* [32]. Rabdosiin and its monosodium salt identified in both OE and OL, as well as its disodium salt detected in OL, have been previously isolated and structurally determined by our scientific group in species of *Alkanna* and *Rindera* [3,4,52]. Similarly, the identification of trigonotins A, B, C and their isomers identified in OG and OE, respectively, have been isolated from *Trigonotis peduncularis* [33], while this is their first detection in *Onosma* genus.

Several derivatives of quinic acid, primarily consisting of esters formed between quinic acid and coumaric or caffeic acids, have been predominantly linked to metabolites derived only from OG, while chlorogenic acid and isomers of methyl caffeoylquinate were identified in both OG and OL. Although chlorogenic acid has been reported in various *Onosma* species such as *O. isaurica*, *O. bracteosa*, *O. ambigens*, *O. aucheriana*, etc. [6], and p-coumaroyquinic acid has been characterized in *O. heterophylla* [49], isomers of methylcaffeoylquinate are reported for the first time, in the current study.

A small group of phenolic compounds identified exclusively in OL comprises derivatives of phenylethanoid glycosides. Through manual dereplication and GNPS annotation, verbascoside (also known as acteoside), characterized by an *m*/*z* ([M-H]^−^) value of 623, was identified as a phenylethanoid triglycoside previously reported in *O. sericea* and *O. aucheriana* [25,56]. By analyzing spectral similarities and correlating them with the literature spectral data, nodes associated with verbascoside exhibiting *m*/*z* ([M-H]^−^) values of 755 and 785 were tentatively annotated as lavandulifolioside and teupolioside, respectively. The feature with an *m*/*z* value of 799 also corresponds to a phenylethanoid triglycoside derivative. These metabolites consist of a phenylethyl alcohol backbone, such as hydroxytyrosol, linked to three glycosyl moieties and a hydroxycinnamic acid group, such as caffeic or coumaric acid [57]. Phenylethanoid glycosides are prevalent in various medicinal plants and are particularly found in families such as Asteraceae, Berberidaceae, Lamiaceae, Plantaginaceae, and Verbenaceae [58]. They exhibit a range of significant bioactivities, including antimicrobial, antitumor, anti-inflammatory, neuroprotective, antioxidant, hepatoprotective, and immunomodulatory effects [58]. Beyond verbascoside, these compounds represent novel phenylethanoid triglycoside derivatives within the genus *Onosma*.

Hydroxybenzoic acids together with vanillic acid and 3,4-dihydroxyphenylacetic acid were present in all examined species, while one dihydroxybenzoic acid was identified in OE. The literature indicates that 3- and 4-hydroxybenzoic acids have been reported in several *Onosma* species, including O*. papillosa*, *O. ambigens*, *O. pulchra*, *O. frutescens*, *O. aucheriana*, *O. sericea*, *O. gigantea*, *O. bracteatum*, *O. isaurica*, *O. bracteosa*, *O. armenum* and *O. inexspectata* [6]. Dihydroxybenzoic acids, such as protocatechuic acid and gentisic acid, as well as the methoxy derivative vanillic acid, have also been identified in many of these species, while 3,4-dihydroxyphenylacetic acid has been reported in *O. pulchra*, *O. frutescens*, *O. aucheriana*, and *O. sericea* [56,59].

Four stilbene derivatives were manually dereplicated and identified as isomers of salvianolic acids F, and salvianolic acid A. Salvianolic acid A has been documented in *Rindera graeca* [52] while salvianolic acid F has been documented in species *Anchusa officinalis* [30]; however, it is reported for the first time within the *Onosma* genus.

Few coumarin derivatives were identified in the studied *Onosma* species. The methoxy coumarin esculetin was present in OE and OG, while all three species shared hydroxycoumarins and one dimethoxycoumarin. Among the genus *Onosma*, only *O. bracteatum* has been reported to be rich in coumarins, among which are 3-hydroxycoumarin, umbelliferone, scopoletin, 6,7-dimethoxycoumarin, and esculetin [32].

**Figure 3:**
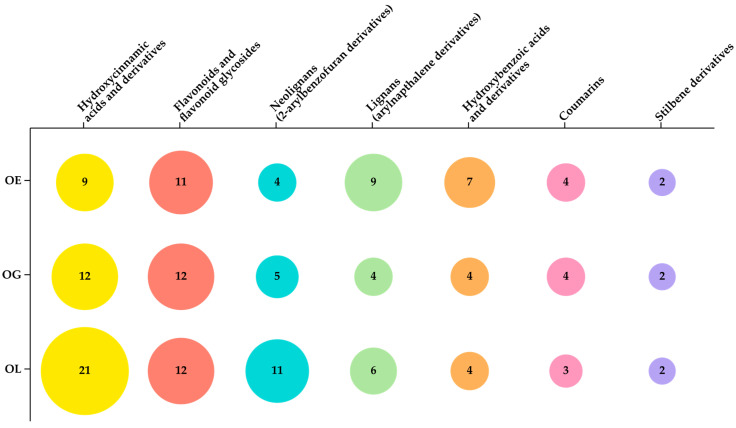
A customized matrix plot diagram illustrates the distribution of dereplicated compounds for each examined species categorized by phenolic class. The size of the circles indicates the number of dereplicated compounds.

The examined *Onosma* species displayed diverse phenolic profiles, with a limited common number of metabolites such as vanillic acid, 3,4-dihydroxyphenylacetic acid, p-coumaric acid, caffeic acid, methyl caffeate, caffeoyl-4′-hydroxyphenyllactic acid, rosmarinic acid, rosmarinic acid methyl ester, rosmarinic acid hexoside, lithospermic acid, hydroxycoumarin, and dimethoxy-coumarin. The visualization of the phenolic profiles through the FBMN revealed that OG exhibited the highest distribution of flavonoid glycosides, whereas OL the highest distribution of hydroxycinnamic and neolignan derivatives. Additionally, OE was characterized by a significant abundance of lignans. Figure 3 illustrates the number of dereplicated compounds for each examined species, which appears consistent with the observations.

### 3.2. Biological Evaluation

The methanolic extracts of *Onosma* species were evaluated for their TPC and TFC. The results of these evaluations are summarized in Table 2, Figure 2A. A significantly higher phenolic content was observed in OL, with a value of 69.03 mg GAE/g extract, while OE and OG exhibited similar phenolic contents of 20.04 and 24.37 mg GAE/g extract, respectively. In contrast, the TFC assessment revealed a greater concentration of flavonoids in OG, with a value of 45.80 mg RE/g extract. OL showed a TFC of 27.94 mg RE/g extract, whereas OE exhibited the lowest value of 5.82 mg RE/g extract. As previously mentioned, both the FBMN and chemical dereplication revealed a substantial variety of hydroxycinnamic acids and neolignan derivatives, particularly in OL, while OG was found to be rich in various flavonoid glycosides, which appears to be consistent with the quantification values.

The antioxidant activities of the methanolic extracts of *Onosma* species were assessed using various methodologies to elucidate potential multiple mechanisms of antioxidant action (Table 3, Figure 2B). Specifically, we employed the DPPH and ABTS assays to evaluate radical scavenging abilities, while the phosphomolybdenum, CUPRAC, and FRAP assays were utilized to determine reducing power potential. Additionally, a metal-chelating assay technique was implemented.

According to the data presented in the figure, OL exhibited the highest antioxidant activity across all assays conducted, with determined values of 230.60 mg TE/g extract for DPPH, 507.77 mg TE/g extract for ABTS, 385.77 mg TE/g extract for CUPRAC, and 501.91 mg TE/g extract for FRAP. The phosphomolybdenum assay yielded a value of 2.11 mmol TE/g extract, while the metal-chelating assay showed an activity of 8.90 mg EDTAE/g extract. The observed superior ability of OL can be attributed to a higher concentration of phenolics in the extract. In contrast, OE and OG displayed lower antioxidant activities; however, when comparing these two species, OE demonstrated higher radical scavenging activity in the DPPH and ABTS assays, whereas OG exhibited superior reducing power in the FRAP, CUPRAC, and phosphomolybdenum assays.

Within each species, a consistent pattern was observed in the antioxidant activity across different assays. All species demonstrated higher activity in the ABTS assay compared to the DPPH assay. Although both assays are employed to assess the in vitro antiradical activity of samples, the radicals utilized in each assay differ structurally [60]. The ABTS assay is regarded as more effective for evaluating complex mixtures that contain hydrophilic, lipophilic, and highly pigmented antioxidant compounds, whereas the DPPH assay is specifically tailored for hydrogen-donating antioxidants [60]. Additionally, differences were noted between the assays used to measure the reducing power of the extracts. The CUPRAC assay employs a copper (II)-containing chromogenic reagent that undergoes a redox reaction with antioxidants present in the sample, the FRAP test assesses the ability of antioxidants to reduce ferric ions (III) to ferrous ions (II), while the phosphomolybdenum assay is based on the reduction of Mo (VI) to Mo (V) from the antioxidants present in the samples [61]. In this study, all extracts exhibited a greater capacity to reduce molybdenum ions over ferric and copper ions.

The metal-chelating assay is a method designed to evaluate the capacity of extracts to bind free ferrous ions, which can catalyze the generation of reactive oxygen species. Notably, OG showed no activity in the metal-chelating assay, while OE’s activity (1.28 mg EDTAE/g extract) was significantly lower than that of OL. Phenolic compounds and flavonoids are known to possess functional groups within their structures that can effectively bind free metal ions [62]. In particular, α-hydroxy carbonyl groups, carboxylate groups, and catecholate moieties found in flavonoids and hydroxycinnamic acid derivatives contribute significantly to their metal-chelating activity [62]. The high concentration of phenolic compounds and especially the presence of flavonols over flavons as well as the numerous caffeic acid derivatives in OL may contribute to its superior metal-chelating activity compared to the other studied extracts.

The enzyme-inhibitory effects of *Onosma* species were evaluated against acetylcholinesterase (AChE) and butyrylcholinesterase (BChE), enzymes implicated in neurodegenerative disorders such as Alzheimer’s disease, as well as α-amylase and α-glucosidase, which are involved in carbohydrate digestion and associated with metabolic disorders like diabetes. These assessments were conducted using spectrophotometric assays, and the results are shown in Table 4, Figure 2C.

The data presented indicate that the inhibitory effects of acetylcholinesterase (AChE) and butyrylcholinesterase (BChE) varied among the extracts. All the extracts exhibited higher AChE inhibitory activity than BChE. OG exhibited the highest inhibition of AChE, with a value of 2.35 mg GALAE/g, followed by OE and OL at 1.99 mg GALAE/g and 1.90 mg GALAE/g, respectively. Conversely, OE demonstrated the strongest inhibition against BChE, with a value of 1.43 mg GALAE/g extract, followed by OL at 1.24 mg GALAE/g and OG at 0.72 mg GALAE/g.

Numerous extracts from *Onosma* species have been investigated for their potential cholinesterase (ChE) inhibitory effects. In recent studies, molecular docking analysis has been utilized to investigate the molecular interaction of compounds from *Onosma* sp. with the ChE enzymes. In these studies, it was found that the binding affinity of compounds including hyperoside and rosmarinic acid from *O. sieheana* and *O. stenoloba* [63] as well as flavonoid glycosides (luteolin 7-glucoside and apigenin 7-glucoside) from *O. bourgaei* and *O. trachytricha* exhibited noticeable binding affinity on the AChE and BChE [64]. Additionally, a recent study identified phenolic compounds in the methanolic extract of *O. riedliana* that were positively correlated with the inhibition of acetylcholinesterase (AChE), but not with butyrylcholinesterase (BChE) [65]. The phenolic acids associated with this correlation included protocatechuic, chlorogenic, 3,4-dihydroxyphenylacetic, vanillic, caffeic, rosmarinic and p-coumaric acids, as well as the flavonoid luteolin-7-glucoside [65]. All of the aforementioned metabolites were also detected in the present analysis. Although we could not establish a statistical relationship between the AChE, and BChE inhibitory activities of the extracts and isolated compounds, their presence in the extracts may contribute to the observed inhibition of cholinesterase enzymes.

In the assays evaluating the inhibitory activities of the extracts on digestive enzymes, a distinct activity profile was observed compared to those obtained for ChE. The data indicate that the inhibitory effects of the extracts on α-amylase are considerably lower than those observed for α-glucosidase. OL demonstrated the strongest inhibition against both α-glucosidase and α-amylase, with values of 5.69 and 0.48 mmol ACAE/g extract, respectively. This was followed by OG, which exhibited values of 3.08 and 0.38 mmol ACAE/g extract, while OE showed the lowest activity, with values of 1.28 and 0.36 mmol ACAE/g extract for α-glucosidase and α-amylase, respectively.

Research has demonstrated the in vitro antidiabetic properties of various *Onosma* species, particularly through their inhibitory effects on the enzymes α-amylase and α-glucosidase. Molecular docking studies on phenolic compounds extracted from *Onosma* species have identified significant binding affinities for hyperoside, luteolin 7-glucoside, and apigenin 7-glucoside with both α-amylase and α-glucosidase enzymes [14,64]. Additionally, a study by Ćavar Zeljković et al. established a positive correlation between the inhibition of α-glucosidase and the presence of 3,4-dihydroxyphenylacetic acid, which was consistently found across all examined extracts [65].

The total phenolic and flavonoid contents, along with the results of the biological assays of the extracts, were analyzed using Pearson correlation analysis. The obtained correlations are presented in Table 5. According to the correlation coefficients, a strong positive correlation was observed between TPC and antioxidant activity assays, as well as between α-amylase and α-glucosidase assays. Specifically, correlation coefficients between phenolics and the DPPH, ABTS, FRAP, and CUPRAC assays were found to be greater than 0.99 (*p* < 0.01), while for the phosphomolybdenum and metal-chelating assays, the coefficients were greater than 0.97 (*p* < 0.01). These findings are significantly consistent with the literature data [56]. A high correlation was also observed between TPC and digestive enzyme inhibition assays, with coefficients of 0.972 (*p* < 0.01) for α-amylase and 0.940 (*p* < 0.01) for α-glucosidase. In contrast, an inverse relationship was observed between the TPC of the extracts and their cholinesterase inhibitory activities; this type of correlation has also been reported previously, in extracts of *Onosma* spp. [56].

Even though high flavonoid content in extracts is typically associated with increased antioxidant activity, no such correlation resulted from the statistical analysis. Therefore, the observed bioactivity of the extracts is likely attributed more to their phenolic content rather than flavonoids. However, quantifying isolated compounds and investigating their biological activities would provide a more comprehensive understanding of the relationship between phytochemical quantities and their exhibited bioactivity.

## 4. Materials and Methods

### 4.1. Plant Material and Methanolic Extract Preparation

The aerial parts of OE, OG and OL were collected from southern Greece during flowering season. Plant materials were botanically identified by Dr. E. Kalpoutzakis (Dept. of Pharmacy, NKUA) (OE, OG) and Prof. T. Constantinidis (Dept. of Biology, NKUA) (OL). Subsequently, they were deposited at the Herbarium of the Laboratory of Pharmacognosy Department of Pharmacy of the National and Kapodistrian University of Athens (NKUA). Collection data for each species are summarized below.

- OE: “Lefka Ori” Mountains, Western Crete; May 2019.
- OL: Taygetos Mountain, Peloponnese; July 2018.
- OG: Parnon Mountain, Peloponnese; June 2019.

The aerial parts of the plants were naturally dried at room temperature in a shaded and well-ventilated environment. After the drying process, the plant materials were ground using a laboratory mill and the grounded materials were then subjected to extraction with methanol for a duration of 24 h. The extraction procedure was repeated three times for each sample. Subsequently, the solvent was removed under vacuum conditions.

### 4.2. High Performance Liquid Chromatography–Mass Spectrometry (UPLC-HRMS) Analysis

Methanolic extracts were analyzed using UPLC-HRMS (Supplementary Materials Figure S1). This was accomplished with an Agilent 6210 system, which includes an HP 1200 chromatograph featuring an autosampler, a binary pump, a thermostated column compartment, and a membrane degasser. The mass spectrometry component consists of a 6210 LC/MSD mass spectrometer equipped with a time-of-flight (TOF) mass analyzer and a dual spray source for electrospray ionization (ESI) to facilitate the analysis of both sample and reference masses. The system is attached to a nitrogen generator (Parker Hannifin Corporation, Haverhill, MA, USA), which produces nitrogen at purities exceeding 99%, as well as a compressed air generator (Jun-Air, Oxymed, Loze, Poland) and a compressed air container. For the chromatographic separation, a Zorbax Stable Bond RP-18 column (250 × 2.1 mm, particle size = 5 μm) was utilized. The mobile phase consisted of system A: 1% MeCN in H2O with 0.1% (*v*/*v*) FA and, 10 mM ammonium formate, (pH = 3.5), and system B: 95% MeCN in H2O with 0.1% (*v*/*v*) FA and, 10 mM ammonium formate, (pH = 3.5). A method with a total run time of 50 min and post time of 15 min was employed: 0–45 min, 1–60% of system B (linear gradient); 45–46 min, 60–90% of system B (linear gradient); 46–50 min, isocratic run 90% of system B. The column temperature was maintained at 25 °C, with a flow rate of 0.2 mL/min and an injection volume of 10 µL. Prior to the analytical procedures, tuning and calibration were conducted using a mixture of ten reference masses. This calibration process ensured that mass measurement errors were maintained at less than 1 ppm. The nitrogen flow rate was set at 10 L/min, with a gas temperature of 350 °C and a pressure of 35 psi. The analysis was executed in negative ionization mode, utilizing various fragmentation voltages of 140 V, 200 V, and 250 V. Data acquisition and analysis were performed using Mass Hunter 2.2.1 LC/MS spectra analysis software.

### 4.3. Molecular Networking and Chemical Dereplication

The raw UPLC-HRMS data files from Agilent, in the *.D format, were first converted to the *.mzML format using the MSConvert tool from the ProteoWizard suite [66,67]. This conversion was necessary for the data processing within the MZMine 4.2.0/MZIO environment (for simplicity, herein after MZMine) [68]. During this conversion, the data underwent centroiding, which involves transforming profile data into a format that captures peak intensities at distinct *m*/*z* values by applying a peak-picking filter. For the data processing in the MZMine environment and the corresponding parameters, consult the supplementary information (Supplementary Materials Table S1).

In order to compare the chemical profiles of the *Onosma* spp. under study using a molecular networking-based approach, the UPLC-HRMS data of all three species were processed simultaneously by employing the exact same workflow and the processing output (a list of aligned features, MS2 spectral data thereof, and sample metadata) was subsequently used for the construction of a feature-based molecular network (FBMN) using the comprehensive Global Natural Products Social Molecular Networking (GNPS2) platform [69,70]. This platform was also used for the tentative annotation of the features (MS2 consensus spectra or nodes) through spectral library matching (expressed as cosine spectral/fragmentation similarity score). The parametrization within the GNPS2 environment included the following general parameters: precursor ion tolerance set to 0.02 Da; fragment ion tolerance set to 0.02. For the networking parameters, the following were chosen: minimum cosine score set to 0.7; minimum matched peaks set to 4. For the library search parameters, the minimum cosine score was 0.7; minimum matched peaks were 4. Lastly, an analogue search was set to enabled. The data from the MZMine were also imported into the robust SIRIUS computational environment [71] to predict the identity and chemical classes of compounds using the built-in tools and algorithms CSI:FingerID [72], CANOPUS [73] and ClassyFire [74]. The outputs from the GNPS2 and SIRIUS analyses were integrated into the Cytoscape environment [75] to enable visual exploration of the extracts’ chemical landscape and to analyze the distribution of metabolites among the species under investigation. For the FBMN layout, the organic style from *yFiles* was selected because it highlights the natural symmetry and clustered structures in undirected graphs, while also promoting a balanced distribution of nodes and reducing edge crossings [yWorks +]. Finally, the FBMN was visually enhanced with the open source InkScape vector graphics editor [The Inkscape Project]. The FBMN output and parametrization of the corresponding workflow can be found on the GNPS2 repository: https://gnps2.org/status?task=a9a85daccf72416486fe75743dc1fc08 (accessed on 1 October 2024).

### 4.4. Total Phenolic Content (TPC) and Flavonoid Content (TFC)

The total phenolic content was determined using the Folin–Ciocalteu method following established procedures [76,77]. A 0.25 mL aliquot of the sample solution was thoroughly mixed with 1 mL of diluted Folin–Ciocalteu reagent (1:9). After a 3 min incubation, 0.75 mL of a 1% Na_2_CO_3_ solution was added. The absorbance of the resulting mixture was measured at 760 nm following a 2 h incubation at room temperature and in darkness. The quantification results were expressed as milligrams of gallic acid equivalents per gram of extract (mg GAE/g extract). For the evaluation of total flavonoid content, the aluminum trichloride (AlCl_3_) method was employed. A 1 mL aliquot of the sample solution was mixed with 1 mL of 2% AlCl_3_ in methanol. A blank control was created by mixing 1 mL of the sample solution with 1 mL of methanol. After incubating for 10 min at room temperature, the absorbances were measured at 415 nm. The results were expressed as milligrams of rutin equivalents per gram of extract (mg RE/g extract).

### 4.5. Biological Assays

#### 4.5.1. Radical Scavenging Activity

The extracts were evaluated for their radical scavenging capacity using two methodologies: the DPPH• (1,1-diphenyl-2-picrylhydrazyl) and the ABTS•+ (2,2′-Azino-bis-(3-ethylbenzothiazoline-6-sulfonic acid)) assays following established procedures [77,78]. Trolox was utilized as a positive control in both assays, and the quantification of the radical scavenging activity of the extracts was expressed as milligrams of Trolox equivalents per gram of extract (mg TE/g extract). In the DPPH• assay, a 1 mL aliquot of the sample solution was combined with 4 mL of a 0.004% DPPH• methanol solution. The absorbance of this mixture was measured at 517 nm following a 30 min incubation in darkness at room temperature. For the ABTS•+ assay, the radical cation was produced by incubating a 7 mM solution of ABTS•+ with 2.45 mM potassium persulfate in the dark at room temperature. The resulting ABTS•+ solution was subsequently diluted with methanol to obtain an absorbance of 0.700 ± 0.02 at 734 nm. Next, 1 mL of the sample solution was mixed with 2 mL of the ABTS•+ solution, and the absorbance was measured at 734 nm, following a 30 min incubation at room temperature.

#### 4.5.2. Reducing Power Assays

The reductive activity of the examined extracts from *Onosma* species was evaluated using the ferric-reducing antioxidant power (FRAP), cupric ion-reducing antioxidant capacity (CUPRAC), and phosphomolybdenum assays as previously described [79,80]. The results were quantified as milligrams of Trolox equivalents per gram of extract (mg TE/g extract) for the FRAP and CUPRAC assays, while the phosphomolybdenum assay results were conveyed in millimoles of Trolox equivalents per gram of extract (mmol TE/g extract). In the FRAP assay, a 0.1 mL aliquot of the sample solution was mixed with a 2 mL solution comprising 2,4,6-tris(2-pyridyl)-s-triazine (TPTZ) at 10 mM in 40 mM HCl, acetate buffer (0.3 M, pH 3.6), and ferric chloride at 20 mM, in a volume ratio of 1:10:1 (*v*/*v*/*v*). The absorbance was measured at 593 nm after incubating for 30 min at room temperature. For the CUPRAC assay, a 0.5 mL sample solution was combined with a mixture of CuCl_2_ (1 mL, 10 mM), neocuproine (1 mL, 7.5 mM), and NH_4_Ac buffer (1 mL, 1 M, pH 7.0). A blank control was produced by mixing 0.5 mL of the sample solution with 3 mL of the aforementioned mixture without CuCl_2_. Absorbance readings were taken at 450 nm after incubating for 30 min at room temperature. In the phosphomolybdenum assay, a 0.3 mL sample solution was combined with a reagent mixture consisting of 3 mL of a solution containing 0.6 M sulfuric acid, 28 mM sodium phosphate, and 4 mM ammonium molybdate. A blank was prepared using 0.3 mL of methanol with the same reagent mixture. The absorbances were recorded at 695 nm after a 90 min incubation at 95 °C.

#### 4.5.3. Metal-Chelating Activity

The metal-chelating activity was assessed using a spectrophotometric method [77], with results quantified as milligrams of EDTA equivalents per gram of extract (mg EDTAE/g extract). A 2 mL aliquot of the sample solution was combined with 0.05 mL of a 2 mM FeCl_2_ solution, followed by the addition of 0.2 mL of a 5 mM ferrozine solution. A blank sample was created using 2 mL of the sample solution, 0.05 mL of FeCl_2_, and 0.2 mL of water, omitting the ferrozine. The absorbances were measured at 562 nm after a 10 min incubation at room temperature.

#### 4.5.4. Enzyme-Inhibitory Activities

The methanolic extracts of the examined *Onosma* species were evaluated for their inhibitory effects against cholinesterases, specifically acetylcholinesterase (AChE) and butyrylcholinesterase (BChE) [80,81], with results quantified in milligrams of galantamine equivalents per gram of extract (mg GALAE/g extract). A 50 μL aliquot of the sample solution was mixed with 125 μL of DTNB (5,5′-dithiobis (2-nitrobenzoic acid)) and 25 μL of AChE or BChE solution in Tris-HCl buffer (pH 8.0). The mixtures were incubated at room temperature for 15 min. Subsequently, the reactions were initiated by adding 25 μL of acetylthiocholine iodide or butyrylthiocholine chloride. Following a further 10 min incubation at room temperature, the absorbances of the mixtures were measured at 405 nm. Additionally, the inhibitory activity of the extracts was assessed against α-glucosidase and α-amylase [82], and the results were quantified in millimoles of acarbose equivalents per gram of extract (mmol ACAE/g extract). In the α-amylase inhibitory assay, a 50 μL of enzyme solution in phosphate buffer (pH 6.9 with 6 mM sodium chloride) was added to a 25 μL sample solution and the mixture was incubated at 37 °C for 10 min. After that, an aliquot of 50 μL of a 0.05% starch solution was added and the mixture was incubated for another 10 min at 37 °C. The reaction was terminated by the addition of 25 μL of 1 M HCl, followed by adding 100 μL of iodine-potassium iodide solution. A blank sample was also prepared for comparison, which did not include α-amylase. The absorbances were measured at 630 nm. For the α-glucosidase inhibitory assay, a 50 μL aliquot of the sample solution was combined with 50 μL of glutathione and 50 μL of α-glucosidase solution in phosphate buffer (pH 6.8), along with 50 μL of PNPG (p-nitrophenyl-α-D-glucopyranoside) and the mixture was incubated for 15 min at 37 °C. For the termination of the reaction 50 μL of sodium carbonate (0.2 M) was added. A blank sample without α-glucosidase was also prepared similarly, and absorbances were recorded at 400 nm.

### 4.6. Expression and Visualization of Results

The aforesaid analyses were performed in triplicate and the results were expressed as mean values and standard deviation (SD). For the investigation of the statistical significance of the differences in the extracts, ANOVA (by Tukey) test (*p* < 0.05) was used and it was performed in Xlstat 2018. Correlation analyses were performed by using a two-tailed Pearson’s correlation test utilizing Graphprism version 8.0.1 software (GraphPad Software, La Jolla, CA, USA), which was also used for the visualization of quantifications and biological assay results. To illustrate manually dereplicated compounds for examined species, we used RAWgraph (rawgraphs.io) [83].

## 5. Conclusions

The current study provides a comprehensive overview of the phenolic profiles of three *Onosma* species growing in Greece—OL, OE, and OG—combining manual and computational approaches as well as evaluating the bioactivities of their methanolic extracts. It is important to emphasize that, to the best of our knowledge, these species have not been investigated in this context before. The study revealed that the examined species contain a diverse array of phenolic compounds, with 94 successfully annotated via manual and computational dereplication approaches. Notably, seven phenolic subgroups were distinguished, including hydroxybenzoic acids, hydroxycinnamic acids, lignans, neolignans, stilbenes, coumarins, and flavonoids. The visualization of the phenolic profiles of the three species through an FBMN, alongside integrated CANOPUS chemical classification predictions, provided significant insight into the similarities and differences between species as well as the distribution of the aforementioned phenolic classes. From the results, it is evident that the phenolic profile of OG is characterized by a dominance of flavonoid glycosides, while the profile of OL is marked by hydroxycinnamic and neolignan derivatives. In contrast, the profile of OE is characterized by a significant abundance of lignans.

This integrated approach led to the annotation of new compounds for the genus *Onosma*, such as several flavonoid glycosides (luteolin- and trihydroxymethoxy-flavone-, isorhamnetin–dihexosides), lignan derivatives (rabdosiin alongside its monosodium and disodium salt, trigonotins A, B, and C) and the stilbenes salvianolic acid A and F. Additionally, several neolignan derivatives of 2-arylbenzofuran subtype are reported for the first time in the genus with tentatively annotated representatives such as methyl lithospermate B, tournefolic acid A, and glycosides of salvianolic acids B and C, as well as the phenylethanoid triglycosides lavandulifolioside and teupolioside.

The quantification of TPC and TFC of the extracts was consistent with the qualitative investigation, showing a high TPC for OL and a high TFC for OG, while the antioxidant assays revealed the strongest activity for the OL extract. The enzyme inhibition assays demonstrated higher AChE inhibitory activity than BChE for all extracts, with OG exhibiting the highest AChE inhibition. In assays evaluating the inhibition of digestive enzymes, all extracts showed greater inhibitory activity against α-glucosidase compared to α-amylase, with the OL extract being the most active. Several annotated compounds appear to have significant binding affinities with the tested enzymes according to the literature [14,63,64,65]. Overall, the potency of the extracts in inhibiting enzymes associated with conditions such as Alzheimer’s disease and diabetes, along with their high antioxidant capacity, highlights the promising therapeutic potential of these plants. Notably, OL demonstrated particularly high inhibition of α-glycosidase, making it a promising candidate for further research in diabetes management.

In conclusion, this study not only enhances our understanding of the chemical diversity within these under-investigated species but also facilitates the identification of novel bioactive compounds with potential ethnopharmacological value. Future studies will aim to expand the dereplication process and uncover other groups of secondary metabolites. This would ultimately provide a more comprehensive understanding of the pharmacological potential of these valuable plants.

## Figures and Tables

**Table 1:** LC/Q-TOF-MS analysis of 3 *Onosma* species’ methanolic extracts.

No.	Rt	[M-H]^−^ *m*/*z*	Molecular Formula	Δppm	MS/MS	Annotated Compounds	Presence in Studied Samples			Determined by
							OE	OG	OL	
Hydroxybenzoic acids and derivatives										
**1.**	5.42	121.0289	C_7_H_6_O_2_	4.08	**121.03**/92.03	Hydroxybenzaldeyde	√			[16]
**2.**	3.91	137.022	C_7_H_6_O_3_	−9.64	93.03	Hydroxybenzoic acid	√	√		[17]
**3.**	14.76	137.0236	C_7_H_6_O_3_	2.04	93.03	Hydroxybenzoic acid	√		√	[17]
**4.**	15.52	137.0237	C_7_H_6_O_3_	2.77	**93.03**/108.02	Hydroxybenzoic acid	√	√	√	[17]
**5.**	5.17	153.0183	C_7_H_6_O_4_	0.42	108.02	Dihydroxybenzoic acid	√			[18]
**6.**	6.11	167.0344	C_8_H_8_O_4_	3.08	152.01/123.02/**108.02**	Vanillic acid	√	√	√	[19]
**7.**	6.67	167.0337	C_8_H_8_O_4_	−1.11	141.48/134.86/**108.02**	3,4-dihydroxyphenylacetic acid	√	√	√	[20]
Hydroxycinamic acids and derivatives										
**8** **.**	7.78	163.039	C_9_H_8_O_3_	0.18	119.05	p-coumaric acid	√	√	√	[19]
**9** **.**	4.26	179.0334	C_9_H_8_O_4_	−2.71	135.04	Caffeic acid	√	√	√	[18]
**10** **.**	15.42	193.0492	C_10_H_10_O_4_	−1.74	161.02/**134.04**/133.03	Methyl caffeate	√	√	√	[21]
**11** **.**	2.25	197.0460	C_9_H_10_O_5_	7.87	135.05/**123.05**/72.99	Danshensu (Salvianic acid A)	√		√	[22]
**12** **.**	5.62	337.0921	C_16_H_18_O_8_	0.91	191.05/173.04/149.91/119.05	Coumaroylquinic acid		√		[23]
**13** **.**	4.56	337.0922	C_16_H_18_O_8_	1.2	261.02/**191.05**/173.04/163.04	Coumaroylquinic acid		√	√	[23]
**14** **.**	24.62	343.0817	C_18_H_16_O_7_	1.37	181.05/**161.02**/135.04	Caffeoyl-4′-hydroxyphenyllactic acid	√	√	√	[24]
**15** **.**	2.96	353.0882	C_16_H_18_O_9_	4.22	**191.05**/179.03/173.04/135.04	Chlorogenic Acid		√	√	[19]
**16** **.**	5.46	367.1025	C_17_H_20_O_9_	0.39	191.05/179.03/173.0.5/161.02/135.04	Methyl caffeoylquinate		√		[23]
**17** **.**	5.02	367.1038	C_17_H_20_O_9_	3.93	191.05/**179.03/**173.05/135.04	Methyl caffeoylquinate			√	[23]
**18** **.**	18.65	359.0753	C_18_H_16_O_8_	−2.35	197.05/179.04/161.02/135.05	Rosmarinic acid	√	√	√	[22]
**19** **.**	29.56	373.0917	C_19_H_18_O_8_	−0.25	193.05/**179.03/**135.04	Rosmarinic acid methyl ester	√	√	√	[24]
**20** **.**	16.2	417.0819	C_20_H_18_O_10_	0.66	373.09/197.04/**175.04**	Salvianolic acid D or isomer			√	[24]
**21** **.**	14.17	521.1296	C_24_H_26_O_13_	1.21	**359.09**/197.04	Rosmarinic acid hexoside			√	[22]
**22** **.**	12.87	521.13	C_24_H_26_O_13_	1.98	463.09/**359.09/**323.07/161.02	Rosmarinic acid hexoside	√	√	√	[22]
**23** **.**	14.03	521.13	C_24_H_26_O_13_	1.98	**359.09**/197.04/135.04	Rosmarinic acid hexoside	√	√		[22]
**24** **.**	9.75	623.1963	C_29_H_36_O_15_	−1.2	623.19/463.17/292.04/161.02	Verbascoside			√	[25]
**25.**	13.53	623.1964	C_29_H_36_O_15_	−1.04	**623.19**/461.16/161.02	Verbascoside isomer			√	[25]
**26.**	12.82	755.2382	C_34_H_44_O_19_	−1.46	**755.24**/594.22/461.18/375.07	Lavandulifolioside isomer			√	[26]
**27.**	8.2	755.2393	C_34_H_44_O_19_	−0.01	**755.24**/593.21/371.05/161.02	Lavandulifolioside			√	[26]
**28.**	12.24	785.2477	C_35_H_46_O_20_	−2.76	**785.22**/623.21/161.02	Teupolioside isomer			√	[27]
**29.**	7.5	785.2504	C_35_H_46_O_20_	0.67	**785.22**/623.21/161.02	Teupolioside			√	[27]
**30.**	13.87	799.2638	C_36_H_48_O_20_	−2.15	**799.27**/623.22/352.02/193.05	Phenylethanoid tryglycoside derivative			√	[28]
**31.**	14.3	799.2643	C_36_H_48_O_20_	−1.53	**799.26**/624.23/526.10	Phenylethanoid tryglycoside derivative			√	[28]
Neoignans derivatives (2-arylbenzofurans)										
**32** **.**	15.24	311.0553	C_17_H_12_O_6_	0.92	**267.07**/130.96	Tournefolic acid A or isomer	√		√	[29]
**33** **.**	28.47	491.0964	C_26_H_20_O_10_	−1.78	**311.05**/295.06/185.02/135.04/109.03	Salvianolic acid C		√	√	[22]
**34** **.**	27.99	491.0967	C_26_H_20_O_10_	−1.17	491.10/**311.005**/135.04	Salvianolic acid C isomer	√			[22]
**35** **.**	9.66	537.1022	C_27_H_22_O_12_	−1.03	493.11/**339.05**/295.06/197.04/135.04	Lithospermic acid	√	√	√	[22]
**36** **.**	7.21	537.1029	C_27_H_22_O_12_	0.27	493.13/**339.05**/295.06/267.06/229.01/197.04/179.04/135.04	Lithospermic acid isomer		√		[22]
**37** **.**	8.77	537.1031	C_27_H_22_O_12_	0.65	494.013/**339.05**/295.06/197.04/179.04	Lithospermic acid isomer			√	[22]
**38** **.**	22.55	653.1508	C_32_H_30_O_15_	1.08	**491.10**/311.05	Salvianolic acid C hexoside or isomer		√	√	
**39** **.**	25.04	715.1306	C_36_H_28_O_16_	1.73	517.08/**337.04**	Dideydrolithospermic acid B			√	[30]
**40** **.**	16.37	717.1421	C_36_H_30_O_16_	−4.06	475.10/**339.05**/243.03/109.03	Salvianolic acid B (Lithospermic acid B)			√	[22]
**41** **.**	21.55	717.1474	C_36_H_30_O_16_	3.33	519.09/**475.11**/339.05/321.05/295.06	Salvianolic acid B isomer			√	[22]
**42** **.**	31.85	731.1582	C_37_H_32_O_16_	−3.37	**533.10**/321.04	Methyl lithospermate B/isomer			√	[24]
**43** **.**	20.87	731.1597	C_37_H_32_O_16_	−1.31	533.11/**489.12/**353.06/135.05	Methyl lithospermate B/isomer	√		√	[24]
**44** **.**	24.67	731.1608	C_37_H_32_O_16_	0.19	**731.15**/551.12/533.11/338.05/229.01	Methyl lithospermate B/isomer		√		[24]
**45** **.**	20.46	879.1984	C_42_H_40_O_21_	0.64	**681.14**/519.08/483.09/321.04	Salvianolic acid B O-hexoside			√	[24]
Lignans derivatives (arylnapthalenes and dihydronapthalenes)										
**46** **.**	9.66	491.0965	C_26_H_20_O_10_	−1.57	491.10/**311.005**/197.04/135.04	Globoidnan A	√	√		[31]
**47** **.**	16.82	537.1021	C_27_H_22_O_12_	−1.21	**339.05**/295.06/197.05	Globoidnan B	√		√	[31]
**48** **.**	29.12	551.1173	C_28_H_24_O_12_	−2	353.06/**321.04**/293.3	9′-Methoxyl salvianolic acid R	√		√	[32]
**49** **.**	17.12	717.1415	C_36_H_30_O_16_	−4.9	519.09/**475.10**/359.08/339.05/197.04/161.03/135.04	Rabdosiin isomer	√			[31]
**50** **.**	27.46	717.1423	C_36_H_30_O_16_	−3.78	**519.09/321.04**	Rabdosiin	√		√	[31]
**51** **.**	7.96	721.1942	C_33_H_38_O_18_	−4.49	**721.19**/677.21/513.13/485.10/397.08	Trigonotin C isomer	√			[33]
**52** **.**	7.83	721.1972	C_33_H_38_O_18_	−0.33	**721.20/**677.11/631.11/557.15/493.22/273.04	Trigonotin C		√		[33]
**53** **.**	13.45	733.1980	C_34_H_38_O_18_	0.76	**733.19**/690.22/367.08/265.04	Trigonotin B	√			[33]
**54** **.**	13.29	733.1948	C_34_H_38_O_18_	−3.60	733.19/**367.08**/229.01	Trigonotin B isomer		√		[33]
**55** **.**	27.27	739.1265	C_36_H_29_O_16_Na	−0.62	739.12/**559.08/**515.05/335.06	Monosodium salt of rabdosiin	√		√	[4]
**56** **.**	26.55	761.107	C_36_H_28_O_16_Na_2_	−2.5	581.07/401.02	Disodium salt of rabdosiin			√	[4]
**57** **.**	13.54	763.2028	C_35_H_40_O_19_	−6.82	**763.20**/749.19/513.13/369.10	Trigonotin A		√		[33]
**58** **.**	13.65	763.2087	C_35_H_40_O_19_	0.91	**763.21/**647.02/369.08/101.02	Trigonotin A isomer	√			[33]
**59** **.**	20.5	983.2819	C_47_H_52_O_23_	0.34	**837.22**/793.25/640.54/367.08/267.08/145.03	Pulmonarioside C			√	[32]
Stilbene derivatives										
**60.**	18.68	313.0687	C_17_H_14_O_6_	−6.28	161.02/133.03/123.04	Salvianolic acid F or isomer		√		[30]
**61.**	17.9	313.07	C_17_H_14_O_6_	−2.12	238.14/**161.02**/133.03/123.04	Salvianolic acid F or isomer		√	√	[30]
**62.**	18.05	313.0706	C_17_H_14_O_6_	−0.21	161.0.2/133.03/123.04	Salvianolic acid F or isomer	√			[30]
**63.**	21.71	493.1123	C_26_H_22_O_10_	−1.26	**295.06**/186.03/135.04	Salvianolic acid A	√		√	[22]
Coumarins										
**64.**	18.04	161.0229	C_9_H_6_O_3_	−2.61	133.03	Hydroxy coumarin		√		[34]
**65.**	17.93	161.0231	C_9_H_6_O_3_	−1.37	133.03	Hydroxy coumarin	√		√	[34]
**66.**	18.68	161.0234	C_9_H_6_O_3_	0.49	**133.03**/104.03	Hydroxy coumarin	√	√	√	[34]
**67.**	3.98	177.0165	C_9_H_6_O_4_	−9.8	135.04	Esculetin	√	√		[35]
**68.**	30.09	205.0494	C_11_H_10_O_4_	−0.66	161.02/133.03	Dimethoxy-coumarin	√	√	√	[30]
Flavonoids and flavonoid glycosides										
**69** **.**	32.3	269.045	C_15_H_10_O_5_	2.04	227.03/151.02/149.02/117.03	Apigenin	√	√		[34]
**70** **.**	28.84	285.0392	C_15_H_10_O_6_	−0.58	175.04/151.00/133.03/107.02	Luteolin	√	√		[36]
**71** **.**	32.66	299.0544	C_16_H_12_O_6_	−2.05	284.03	Diosmetin		√		[37]
**72** **.**	17.51	431.096	C_21_H_20_O_10_	−2.95	269.04	Apigenin-O-hexoside	√			[38]
**73** **.**	16.81	431.0969	C_21_H_20_O_10_	−0.87	269.04	Apigenin-7-*O*-glucoside		√	√	[39]
**74** **.**	11.6	447.0903	C_21_H_20_O_11_	−4.22	285.04	Luteolin-7-*O*-glucoside		√		[39]
**75** **.**	17.62	447.091	C_21_H_20_O_11_	−2.66	285.04	Kaempferol-3-*O*-glucoside	√			[40]
**76** **.**	16.5	447.0917	C_21_H_20_O_11_	−1.09	284.03/255.03/227.03	Kaempferol-3-*O*-galactoside	√		√	[40]
**77** **.**	12.24	463.0873	C_21_H_20_O_12_	0.43	301.03	Quercetin-3-*O*-galactoside	√		√	[40]
**78** **.**	11.59	463.0875	C_21_H_20_O_12_	0.86	300.03	Quercetin-3-*O*-glucoside	√		√	[40]
**79** **.**	16.33	577.152	C_27_H_30_O_14_	−5.51	269.04	Apigenin-*O*-hexose-*O*-deoxyhexose		√		[41]
**80** **.**	14.89	577.1522	C_27_H_30_O_14_	−5.17	459.11/**269.04**	Apigenin-*O*-hexose-*O*-deoxyhexose		√		[41]
**81** **.**	8.25	593.1487	C_27_H_30_O_15_	−2.35	**285.04**/133.03	Luteolin-7-*O*-hexose-*O*-deoxyhexose		√		[19]
**82** **.**	9.25	593.1488	C_27_H_30_O_15_	−2.19	285.04	Luteolin-7-*O*-rutinoside		√		[42]
**83** **.**	15.01	593.1491	C_27_H_30_O_15_	−1.68	**593.15**/549.29/417.25/285.04/151.00	Kaempferol-*O*-hexose-*O*-deoxyhexose	√		√	[41]
**84** **.**	14.63	593.1507	C_27_H_30_O_15_	1.02	**593.15**/285.04	Kaempferol-*O*-hexose-*O*-deoxyhexose	√			[41]
**85** **.**	16.41	607.1609	C_28_H_32_O_15_	−7.98	607.16/**299.05**	Diosmetin-rhamnosylglucoside (diosmin)		√		[43]
**86** **.**	10.07	609.1434	C_27_H_30_O_16_	−2.65	301.03/300.03	Rutin	√		√	[39]
**87** **.**	6.54	609.1438	C_27_H_30_O_16_	−1.99	447.09/301.03/285.03	Quercetin-*O*-hexoside-*O*-deoxyhexose		√		[44]
**88** **.**	9.34	609.1447	C_27_H_30_O_16_	−0.51	301.03/300.03/151.00	Quercetin-*O*-hexoside-*O*-deoxyhexose	√		√	[44]
**89** **.**	7.38	609.1447	C_27_H_30_O_16_	−0.51	541.03/343.04/301.03/243.04/154.11	Quercetin-*O*-hexoside-*O*-deoxyhexose		√		[44]
**90** **.**	22.96	623.1635	C_28_H_32_O_16_	4.56	461.11/**299.05**	Trihydroxy-methoxyflavone dihexoside			√	[45]
**91** **.**	15.71	639.1553	C_28_H_32_O_17_	−0.43	477.10/**315.05/**300.02/285.03/181.05	Isorhamnetin dihexoside			√	[46]
**92** **.**	20.08	651.1564	C_29_H_32_O_17_	1.27	**651.16**/429.07/285.04	Luteolin acetyl-dihexoside			√	[47]
**93** **.**	30.04	665.1696	C_30_H_34_O_17_	−2.44	299.05	Trihydroxy-methoxyflavone acetyl-dihexoside			√	[48]
**94** **.**	20.98	681.168	C_30_H_34_O_18_	2.73	639.16/315.05/300.02/135.05	Isorhamnetin acetyl-dihexoside			√	[46]
Total annotations for each species							**46**	**43**	**59**	

The most abundant ions are shown in bold.

**Table 2:** Total phenolic and total flavonoid contents of methanol extracts from three studied *Onosma* species *.

Phytochemical Assays	OE	OG	OL
Total phenolic content (mg GAE/g extract)	20.04 ± 0.22 ^a^	24.37 ± 1.81 ^b^	69.03 ± 1.68 ^c^
Total flavonoid content (mg RE/g extract)	5.82 ± 0.08 ^a^	45.80 ± 0.40 ^b^	27.94 ± 0.40 ^c^

* Values expressed are means ± S.D. of three parallel measurements. GAE. gallic acid equivalents; RE. rutin equivalents. Different superscripts in the same raw indicate significant difference (*p* < 0.05).

**Table 3:** Antioxidant properties of methanol extracts from three studied *Onosma* species *.

Antioxidant Assays	OE	OG	OL
DPPH• (mg TE/g extract)	63.36 ± 1.15 ^a^	59.16 ± 1.69 ^a^	230.60 ± 2.74 ^b^
ABTS•+ (mg TE/g extract)	136.40 ± 1.32 ^a^	123.50 ± 1.49 ^a^	507.77 ± 9.36 ^b^
FRAP (mg TE/g extract)	111.21 ± 0.76 ^a^	122.22 ± 1.78 ^b^	385.77 ± 3.47 ^c^
CUPRAC (mg TE/g extract)	124.82 ± 4.80 ^a^	145.29 ± 3.88 ^b^	501.91 ± 8.31 ^c^
Phosphomolybdenum (mmol TE/g extract)/ (mgTE/g extract) **	1.07 ± 0.05 ^a^ (267.32 ± 12.54) **	1.11 ± 0.03 ^a^ (277.43 ± 7.51) **	2.11 ± 0.16 ^b^ (528.65 ± 40.05) **
Metal chelating (mg EDTAE/g extract)	1.28 ± 0.16 ^a^	na	8.90 ± 0.21 ^b^

* Values expressed are means ± S.D. of three parallel measurements. TE. trolox equivalents; EDTAE: EDTA equivalents; na: not active. Different superscripts in the same raw indicate significant difference (*p* < 0.05). ** Converted values (mg TE/g extract) from original measurements (mmol TE/g extract).

**Table 4:** Enzyme-inhibitory activity of methanol extracts from three studied *Onosma* species *.

Enzyme-Inhibitory Assays	OE	OG	OL
AChE Inhibition (mg GALAE/g extract)	1.99 ± 0.03 ^a^	2.35 ± 0.11 ^b^	1.90 ± 0.20 ^a^
BChE Inhibition (mg GALAE/g extract)	1.43 ± 0.10 ^a^	1.24 ± 0.07 ^a^	0.72 ± 0.06 ^b^
α-amylase inhibition (mmol ACAE/g extract)/ (mg ACAE/g extract) **	0.36 ± 0.02 ^a^/ (232.42 ± 12.87) **	0.38 ± 0.01 ^a^/ (244.69 ± 6.46) **	0.48 ± 0.01 ^b^/ (309.41 ± 6.46) **
α-glucosidase inhibiton (mmol ACAE/g extract)/(mg ACAE/g extract) **	1.28 ± 0.13 ^a^/ (825.43 ± 83.82) **	3.08 ± 0.17 ^b^/ (1987.33 ± 109.78) **	5.69 ± 0.06 ^c^/ (3678.67 ± 38.67) **

* Values expressed are means ± S.D. of three parallel measurements. AChE: acetylcholinesterase; BChE: butyrylcholinesterase; GALAE: galanthamine equivalents; ACAE: acarbose equivalents; na: not active. Different superscripts in the same raw indicate significant difference (*p* < 0.05). ** Converted values (mg ACAE/g extract) from original measurements (mmol ACAE/g extract).

**Table 5:** Correlations between total phenolic and total flavonoid contents and biological assays ^a^.

	DPPH	ABTS	FRAP	CUPRAC	Phospho- Molybdenum	Metal Chelating	AChE	BChE	α-Amylase	α-Glucosidase
TPC	0.993 ^b^	0.992 ^b^	0.998 ^b^	0.997 ^b^	0.980 ^b^	0.975 ^b^	−0.493	−0.951 ^b^	0.972 ^b^	0.940 ^b^
TFC	0.040	0.032	0.097	0.109	0.093	−0.072	0.624	−0.310	0.211	0.460

^a^ Data show the Pearson correlation coefficients between the parameters. DPPH: 2,2-Diphenyl-1-picrylhydrazyl, ABTS: 2,2′-azino-bis-3-ethylbenzthiazoline-6sulphonic acid, FRAP: ferric reducing antioxidant power, CUPRAC: cupric-reducing antioxidant capacity, AChE: acetylcholinesterase, BChE: butyrylcholinesterase. ^b^ Significant difference *p* < 0.01

## Data Availability

The original contributions presented in this study are included in the article/Supplementary Material. Further inquiries can be directed to the corresponding author.

## Supplementary Materials

The following supporting information can be downloaded at https://www.mdpi.com/article/10.3390/plants13243468/s1: Figure S1: Base peak chromatograms (BPCs) of (A) *O. erecta*, (B) *O. graeca* (B), (C) *O. leptantha*, samples, acquired in ESI negative ionization mode; Figure S2. Full FBMN of *Onosma* spp. as exported from GNPS2 and visualized in Cytoscape. *O. erecta*–yellow; *O. leptantha*–green; *O. graeca*–blue; Table S1. Parameters applied during data processing in the MZMine4/MZIO workflow. References [71,72,73,78] are cited in the supplementary materials.

## Abbreviations

OE	*Onosma erecta*;
OG	*Onosma graeca*;
OL	*Onosma leptantha*;
AChE	Acetylcholinesterase;
BChE	Butyrylcholinesterase;
DPPH	2,2-diphenyl-1-Picrylhydrazyl;
ABTS	2,2′-azino-bis-3-ethylbenzothiazoline-6-sulfonic acid;
TE	Trolox equivalents;
RE	Rutin equivalents;
GAE	Gallic acid equivalents;
UPLC-HRMS	Liquid chromatography/quadrupole time-of-flight tandem mass spectrometry;
CUPRAC	Cupric ion-reducing antioxidant capacity;
FRAP	Ferric-reducing antioxidant power;
TPC	Total phenolic content;
TFC	Total flavonoid content;
FBMN	Feature-based molecular network;
GNPS	Global Natural Products Social Molecular Networking.
