# Supplementary material for: A Comparative UPLC/HRMS Molecular Networking-Enhanced Study on the Phenolic Profiles and Bioactivities of Three Medicinally Significant Species of Onosma (Boraginaceae)
Source: Plants (Basel). 2024 Dec 11;13(24):3468. doi: 10.3390/plants13243468 (PMC11676079; doi:10.3390/plants13243468)
Supplement: Supplementary file 1 [file plants-13-03468-s001.zip › plants-3332273-supplementary.pdf]

plants

MDPI

Article

A Comparative UPLC/HRMS Molecular Networking-Enhanced Study on the Phenolic Profiles and Bioactivities of Three Medicinally Significant Species of *Onosma* (Boraginaceae)

Supplementary Materials

Figure S1.: Base peak chromatograms (BPC) of **(A)** *O. erecta*, **(B)** *O. graeca* (B), **(C)** *O. leptantha*, samples, acquired in ESI negative ionization mode.

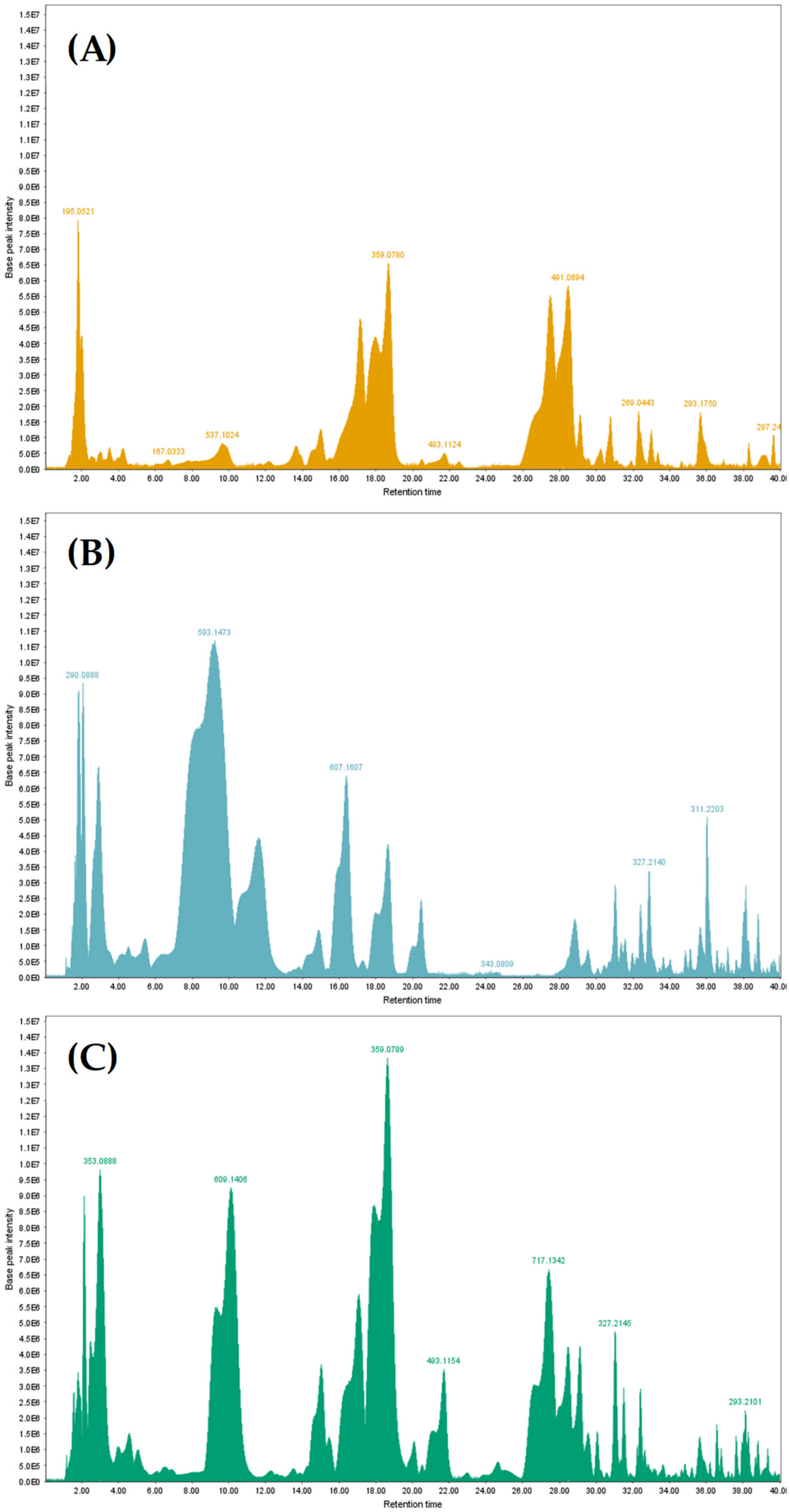

Figure S2. Full FBMN of *Onosma* spp. as exported from GNPS2 [72,73] and visualised in Cytoscape [78]. *O. erecta* – yellow; *O. leptantha* – green; *O. graeca* – blue.

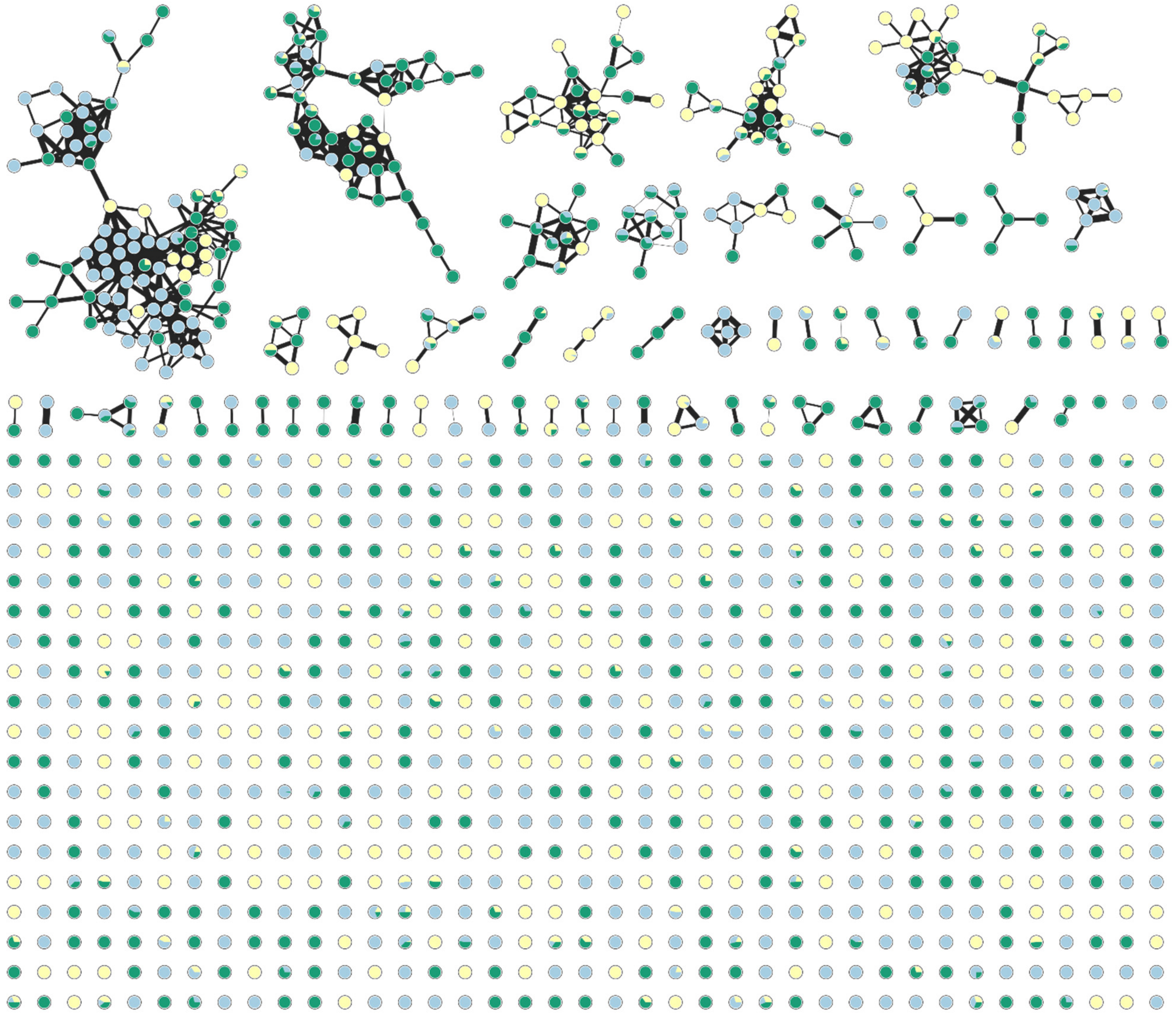

Table S1. Parameters applied during data processing in the MZMine4/MZIO [71] workflow:

**Mass detection**
Scan filters: MS1
Mass detector: Centroid
Noise level: 1500.0
**Mass detection**
Scan filters: MS2
Mass detector: Centroid
Noise level: 100.0
**Chromatogram builder**
Scan filters: MS1
Retention time: [0.05..40.0]

MS level filter: MS1
Polarity: -
Spectrum type: CENTROIDED
Minimum consecutive scans: 5
Minimum intensity for consecutive scans: 1500.0
Minimum absolute height: 10000.0
m/z tolerance (scan-to-scan): 0.01 m/z or 10.0 ppm
**Local minimum feature resolver**
MS/MS scan pairing: true
Dimension: Retention time
Chromatographic threshold: 0.95
Minimum search range RT/Mobility (absolute): 0.1
Minimum relative height: 0.0
Minimum absolute height: 10000.0
Min ratio of peak top/edge: 1.0
Peak duration range (min/mobility): [0.0..5.0]
Minimum scans (data points): 5
**13C isotope filter (formerly: isotope grouper)**
m/z tolerance (intra-sample): 0.01 m/z or 10.0 ppm
Retention time tolerance: 0.02 minutes
Mobility tolerance: false (null)
Monotonic shape: true
Maximum charge: 1
Representative isotope: Most intense
Never remove feature with MS2: true
**Isotope finder module**
Chemical elements: H, C, N, O
m/z tolerance (feature-to-scan): 0.01 m/z or 10.0 ppm
Maximum charge of isotope m/z: 1
Search in scans: SINGLE MOST INTENSE
**Join aligner**
m/z tolerance (sample-to-sample): 0.01 m/z or 10.0 ppm
Weight for m/z: 3.0
Retention time tolerance: 0.1 minutes
Weight for RT: 1.0
**Feature filter**
Keep only features with MS/MS scan: true
